# Serum Biomarkers of Myocardial Remodeling and Coronary Dysfunction in Early Stages of Hypertrophic Cardiomyopathy in the Young

**DOI:** 10.1007/s00246-017-1593-x

**Published:** 2017-03-30

**Authors:** E. Fernlund, T. Gyllenhammar, R. Jablonowski, M. Carlsson, A. Larsson, J. Ärnlöv, P. Liuba

**Affiliations:** 1 Pediatric Heart Center, Skane University Hospital, Lund University, Lund, Sweden; 2 Department of Clinical Sciences, Lund University Hospital, Lund University, Lund, Sweden; 3 Department of Medical Sciences, Uppsala University, Uppsala, Sweden; 4 Department of Medical Sciences, Cardiovascular Epidemiology, Uppsala University, Uppsala, Sweden; 5 Department of Neurobiology, Division of Family Medicine and Primary Care, Care Science and Society, Karolinska Institutet, Huddinge, Sweden; 6 Department of Paediatrics, Linköping University Hospital, Linköping University, Linköping, Sweden

**Keywords:** Hypertrophic cardiomyopathy, Risk, Early stage, Biomarkers, Myocardium

## Abstract

Hypertrophic cardiomyopathy (HCM) remains the leading cause of sudden cardiac death in the young. Early markers for HCM are important to identify individuals at risk. The aim of this study was to investigate novel serum biomarkers reflecting myocardial remodeling, microfibrosis, and vascular endotheliopathy in the early stages of familial HCM in young patients. Twenty-three HCM patients, 16 HCM-risk individuals, and 66 controls (median 15 years) underwent echocardiography and serum analysis for cathepsin S, endostatin, myostatin, type I collagen degradation marker (ICTP), matrix metalloproteinase (MMP)-9, vascular endothelial growth factor receptor (VEGFR)-1, and vascular and intercellular adhesion molecules (VCAM, ICAM). In a subset of the population, global myocardial perfusion was performed by magnetic resonance imaging. Cathepsin S (*p* = 0.0009), endostatin (*p* < 0.0001), MMP-9 (*p* = 0.008), and VCAM (*p* = 0.04) were increased in the HCM group and correlated to left ventricular mass index and mitral E/e′ (*p* < 0.01). In the HCM-risk group, myostatin was decreased (*p* = 0.004), whereas ICAM was increased (*p* = 0.002). Global perfusion was decreased in the HCM group (*p* < 0.05) versus controls. Endostatin and mitral E/e′ correlated inversely to myocardial perfusion (*p* ≤ 0.05). This is the first study demonstrating adverse changes in biomarkers reflecting myocardial matrix remodeling, microfibrosis, and vascular endotheliopathy in early stage of hypertrophic cardiomyopathy in the young.

## Introduction

Hypertrophic cardiomyopathy (HCM) is the most common monogenic cardiac disorder and is one of the leading causes of sudden cardiac death in the young. HCM is characterized by gradual thickening of the cardiac muscle and increased risk for major cardiac events, including ventricular arrhythmias and sudden cardiac death [1–3]. As sarcomere gene mutations can be found in nearly two-thirds of HCM cases [2, 4–6], genetic testing is recommended to identify presymptomatic relatives at risk [2, 4].

The complexity of the disease includes intracellular as well as extracellular changes [7–9].

Postmortem studies of HCM heart specimens have demonstrated myocyte disarray, fibrosis, and remodeling, as well as changes in the coronary circulation, particularly at the microvascular level [7–9]. There is also evidence of increased myocardial extracellular volume in individuals with HCM-causing mutations in the absence of overt hypertrophy [10]. We have recently demonstrated changes in the cardiac diastolic function and peripheral microvascular function in children and young adults with HCM or at risk for this disease [11]. There are limited data regarding the myocardial remodeling process and the timeline of these changes in the early phase of HCM.

Whether these important morphological features can be tracked in the blood of young individuals with HCM has not been yet reported. Cardiac extracellular matrix (ECM) is predominantly composed of type I (85%) and type III (11%) collagen synthesized by cardiac fibroblasts, and plays an important role in myocardial remodeling [12]. The C-terminal telopeptide of collagen type I (ICTP) can be used as a marker of collagen degradation [13]. ICTP undergoes spontaneous denaturation into inactive fragments. Matrix metalloproteinases (MMP) 2 and 9 are part of the enzyme system primarily responsible for ECM degradation and turnover [12]. Elevated serum levels of MMP-2 and MMP-9 have been observed in adult HCM patients [14]. Recently, elevated serum levels of C-terminal telopeptide of collagen type I (ICTP) have been found in adult patients with DCM and HCM [13–16] and also in presymptomatic adults at risk for developing HCM [17]. Cathepsin S is one of the major cysteine proteases, being expressed in the lysosome of the antigen-presenting cells [18]. Cathepsin S is also an important regulator of endostatin [19], which is the C-terminal fragment of collagen XVIII found in the basement membrane zones around blood vessels. Endostatin acts as an antiangiogenic growth factor and is suggested to be synthetized in the coronary circulation in patients with coronary heart disease. It may play an important role in the regulation of coronary angiogenesis [20–22], which is markedly impaired in advanced stages of HCM. Endostatin has also been suggested to be a marker for extracellular matrix remodeling in the heart [23]. Myostatin is a protein derived from the transforming growth factor (TGF)-family, and exerts inhibiting effect on the skeletal muscle cell growth and myogenic differentiation [24]. Myostatin is mainly found not only in skeletal muscle but also in the heart and adipose tissue. Myostatin can be induced in response to cardiac stress, mechanical stretch, or humoral factors such as IGF-1, phenylephrine, or angiotensin II, and has both profibrotic and antihypertrophic actions [24]. Gross muscle hypertrophy has been demonstrated in a pediatric case presenting with loss of function in the myostatin gene [25]. In this case, there was a very low level of circulating myostatin, whereas increased levels of myostatin have been reported in patients with severe heart failure due to ischemic and dilated cardiomyopathy [26] and elevated levels of myostatin are thought to contribute to cardiac cachexia [24].

Intercellular adhesion molecule (ICAM) and vascular cell adhesion molecule (VCAM) are central in the transendothelial migration of inflammatory cells, with a role in endothelial signaling and adhesion as part of the proinflammatory response [27]. Vascular endothelial growth factor receptor 1 (VEGFR1) has long been used as a marker for endothelial function and inflammatory response [28].

The aim of current study was to investigate whether serum biomarkers reflecting myocardial remodeling, microfibrosis, inflammation, and vascular endotheliopathy are affected in the early stage of familial HCM in young individuals.

## Methods

In total 105 participants (median age 15, range 0–30 years) were included in this study. Thirty-nine young individuals with clinical diagnosis of HCM (*n* = 23) and HCM-risk individuals (*n* = 16), all with heredity for familial HCM were recruited from 26 unrelated families with familial HCM from the outpatient clinics of the Pediatric and Adult Cardiology at the Lund University Hospital. Familial HCM was defined as the presence of left ventricular hypertrophy (*Z* score for intraventricular septum (IVS) and/or LV posterior wall (PW) thickness > 2.5 SD on echocardiography) associated with HCM-causing mutation in the family or with heredity for HCM in first-degree relatives. Exclusion criteria were familial HCM with outflow obstruction (HOCM), sporadic cases of HCM (i.e., cases without heredity for familial HCM), or LV hypertrophy (LVH) due to other causes, including congenital heart disease (aortic stenosis, coarctation of the aorta), Noonan syndrome, malformation syndromes, neuromuscular and metabolic disorders, including diabetes, as well as smoking and hypertension.

The HCM-risk group consisted of offspring or siblings to the index patients with HCM. All risk individuals had normal echocardiographic examination and normal 12-lead electrocardiogram (ECG). The control group (*n* = 66) consisted of healthy volunteers. A group of young individuals with endurance physical exercise (athlete group; *n* = 14, training >10 h/week, with left ventricular hypertrophy (LVH, *Z* score > 2 SD)) was also included, and served as LVH controls for HCM patients. Both controls and athletes had 12-lead ECGs in normal range, and no history or heredity of cardiac disease.

All participants and their guardians (for those under 18 years of age) were given verbal and written information, and written consent was obtained. The study was approved by the Regional Ethics Committee at Lund University, Sweden.

All participants underwent physical examination, 12-lead ECG, and echocardiography. On the same occasion, blood samples were taken for later analysis of serum biomarkers of collagen metabolism and degradation, extracellular matrix remodeling, systemic inflammation, and vascular endothelial dysfunction. A subgroup of the cohort also underwent cardiac magnetic resonance imaging (CMR). A registered research nurse collected the demographic data.

### Electrocardiography

Conventional 12-lead resting ECG (Schiller AT-102, Switzerland) was performed in all the participants, and analyzed using pooled standard ECG criteria. LV hypertrophy was assessed according to age-specific conventional ECG criteria that have earlier been described in detail [11].

### Echocardiography

Transthoracic ECG-triggered echo was performed using Philips’ iE33 system (Netherlands) in accordance to the American Society of Echocardiography’s recommendations. The echocardiographic methods have earlier been described in detail [11]. In summary, the LVH was defined as LV wall thickness in end-diastole exceeding +2 SD (in athletes) or +2.5 SD (in HCM group) from the mean corrected for age, gender, and body surface area (BSA). The measurements of LV structures were expressed as *Z* scores (*Z*) in relation to normal distributions adjusted for age and BSA. For individuals older than 18 years, the normal upper limit for IVS or PW was set at 13 mm. LV diastolic function was assessed via pulsed Doppler of the mitral inflow by measuring the early filling (E) peak velocity [29]. Tissue doppler imaging (TDI) was performed to measure the early diastolic (e′) velocity [29]. E/e′ ratio was used as index of LV diastolic filling pressure [29]. LV mass (LVM) was calculated from M-mode measurements, and indexed by dividing it to height raised to exponential power of 2.7. LVM g/m^2.7^ has been earlier shown to reduce the variability among young normal subjects, providing thus a more sensitive cut-off for LVH [30].

### Cardiac Magnetic Resonance Imaging

CMR was performed using a 1.5 T MR scanner (*Philips Achieva*, Philips Healthcare, Best, Netherlands) with a 32-channel coil and ECG triggering. Myocardial perfusion (MP) was measured using flow quantification of the coronary sinus during adenosine-induced hyperemia and at rest. The flow was divided by LVM from cine images as earlier described [31, 32] and validated [33]. MP was expressed in ml/min/g and the MP ratio (MPR) was calculated by dividing MP during adenosine with MP at rest. All CMR analysis was performed using the software Segment [34].

### Biochemical Analysis

Blood samples were obtained from all participants in this study. The analyses were done on EDTA to minimize influence of proteolytic enzymes. The analysis included serum biomarkers of collagen metabolism and degradation, extracellular matrix remodeling, as well as inflammation and endothelial dysfunction, by analysis of ICTP, MMP-9, myostatin, cathepsin S, endostatin, VEGFR1, VCAM, and ICAM.

Endostatin (DY1098), cathepsin S (DY1183), matrix metalloproteinases (MMP-9, DY911), VCAM-1 (DY809), and ICAM-1 (DY720) were analyzed using a commercially available sandwich ELISAs from R&D Systems (Minneapolis, MN, USA), in which a monoclonal antibody specific for the peptide was coated onto microtitre plates. Standards and samples were pipetted into the wells and the peptide was bound to the immobilized antibodies. After washing, a biotinylated antibody was added. After another incubation and washing cycle, a streptavidin-HRP conjugate was added. After incubation and washing, a substrate solution was added. The development was stopped and the absorbance was measured in a SpectraMax 250 (Molecular Devices, Sunnyvale, CA, USA). The peptide concentrations in the samples were determined by comparing the optical density of the sample with the standard curve. ICTP was measured with the UniQ EIA from Orion Diagnostica (Espoo, Finland). The total coefficients of variation (CV) for the assays were in the range of 5–7%. GDF-8/myostatin was analyzed with a Quantikine ELISA from R&D Systems (DGDF80).

Information regarding the presence of the disease-causing mutation and the family history of HCM was obtained from medical records. All HCM genetic analyses, including DNA sequencing and examination of the coding exons of the 11 most common HCM-associated genes *MYH7, MYBPC3, MYL2, MYL3, TNNT2, TPM1, ACTC1, TNNI3, CSRP3, TCAP* and the promoter area of *PLN* in the proband (HCM patient) were performed by Statens Serum Institut (SSI), Copenhagen, Denmark [35].

### Statistical Analysis

Data are presented as mean +/− SEM unless otherwise specified. The differences between the groups were calculated using analysis of variance (ANOVA). Log transformation was used for variables with skewed (non-Gaussian) distribution. When significant, Bonferroni post hoc testing was used to calculate the p values. A result with *p* < 0.05 was considered statistically significant. Logarithmic regression analysis was used to describe the linear relationship between selected variables. All statistical analyses were performed using the statistical software Statview® 5.0 for Windows (SAS Institute Inc., Cary, USA).

## Results

In total, 105 participants (median 15, range 0–30 years) were enrolled in this study as follows: 23 HCM patients, 16 HCM-risk individuals with heredity for familial HCM, and 66 healthy controls, including 14 young athletes. The demographic characteristics of the study population are summarized in Table 1.

**Table 1:** The demographic characteristics of the study groups, data shown as mean ± SEM

	Controls	Athletes	HCM-risk	HCM
*N*	52	14	16	23
Age, y	17.4±0.9	17.5 ± 1.5	12.8 ± 1.6	17.5 ± 1.6
Heredity	0	0	16	14
Boys, *n*	26	13	9	15
Girls, *n*	26	1	7	8
Weight, kg	59.7 ± 2.7	68.0 ± 5.0	48.1 ± 5.2	63.3 ± 5.2
Length, cm	164.1 ± 2.4	173.5 ± 3.4	148.8 ± 6.9	162.1 ± 6.2
Body surface area	1.63 ± 0.04	1.79 ± 0.08	1.39 ± 0.1	1.67 ± 0.1
Body mass index	21.5 ± 0.6	22.1 ± 0.9	20.2 ± 0.8	22.5 ± 1.0
Blood pressure	111/68±1/1	107/71±1/1	107/67±4/1	109/67±3/1

Fourteen of the 23 HCM patients (61%) were carriers of the previously described HCM -causing mutations as follows: Eight patients had *MYBPC3* mutation, four patients had *MYH7* mutation (one HCM patient had double mutation *MYBPC3* and *MYH7)*, one patient had *MYBPC3* mutation and a *MYBPC3* variant, one patient had *TNNT2* mutation, and one patient had a *TCAP* mutation. Twenty-one of the 23 HCM patients were classified as NYHA and two (female, age 7 and 12 years) were classified as NYHA II.

### Echocardiography

The HCM group and the athletes had comparable degree of hypertrophy and left ventricular mass index (*p* > 0.1), whereas the *Z* scores for the LV PW and IVS, and LVM were not significantly different between the control and HCM-risk group (*p* > 0.2). The mitral septal E/e′ was increased in the HCM and HCM-risk groups (*p* < 0.05) compared to controls and athletes. The athletes had significant lower E/e′ even though the degree of LVM was similar versus HCM (*p* = 0.006). The echocardiographic characteristics of the study population are summarized in Table 2.

**Table 2:** The echocardiographic characteristics of the study groups, data shown as mean ± SEM

	Controls	Athletes	HCM-risk	HCM
IVS(mm)	8.3 ± 0.2	10.2 ± 0.5	8.2 ± 0.5	17.6 ± 1.5
IVS(SD)	1.0 ± 0.1	2.3 ± 0.3	1.6 ± 0.3	6.6 ± 0.7
PW(mm)	7.6 ± 0.2	9.1 ± 0.5	7 ± 0.4	10.3 ± 0.7
PW(SD)	−0.16 ± 0.1	0.75 ± 0.5	−0.2 ± 0.4	2.4 ± 0.4
FS(%)	38 ± 0.8	36 ± 0.9	36.2 ± 1.2	44 ± 2
EF(%)	68 ± 1.0	66 ± 1.2	66 ± 1.5	75 ± 2.2
E/e′ septal	7.3 ± 0.4	6.8 ± 0.2	8.3 ± 0.4	10.2 ± 0.8
E/e′ > 8	24%	0	62%	62%
E/e′ > 12	0	0	0	25%
LVM/BSA(g/m^2^)	78.9 ± 2.8	95.9 ± 5.3	72.7 ± 4	130.9 ± 11.8
LVM index(g/m^2.7^)	33.7 ± 0.9	38.7 ± 0.9	34.5 ± 1.9	59.8 ± 6.2

*IVS* intraventricular septum in diastole (mm), *PW* posterior wall in diastole (mm), *FS* fractional shortening (%), *EF* ejection fraction (%), E/e′ septal ratio quotient of mitral inflow E (by pulsed Doppler), and septal e′ measured by tissue Doppler

LVM index represents index of left ventricular mass in relation to the individuals body surface area (in LVM g/m^2^) and in LVM g/m^2.7^

### Biomarkers Reflecting Myocardial Remodeling, Coronary, and Microvascular Dysfunction

**Fig. 1:**
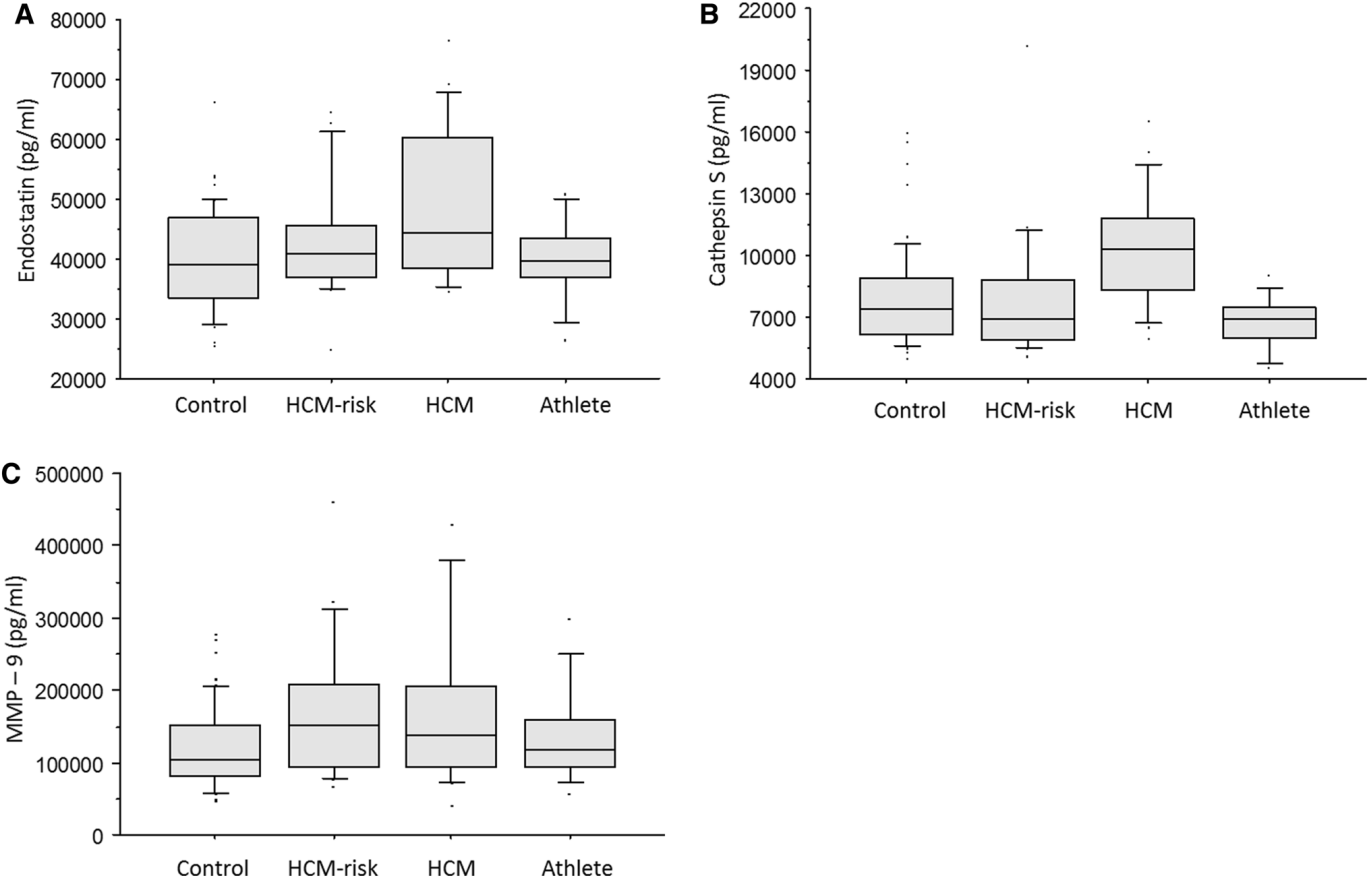
**a** Endostatin was higher in the HCM group than in controls (*p* = 0.0001), athletes (*p* = 0.005), and the HCM-risk group (*p* = 0.03). Data are shown as median and interquartile range. **b** Cathepsin S was higher in the HCM group than in controls (*p* = 0.0009), athletes (*p* = 0.0002), and the HCM-risk group (*p* = 0.02). Data are shown as median and interquartile range. **c** MMP-9 was higher in the HCM group than in controls (*p* = 0.008). There was no statistically significant difference between the HCM group and the other 2 groups (*p* = 0.1 vs. athletes, and *p* = 0.3 vs. the HCM-risk group). Data are shown as median and interquartile range.

**Fig. 2:**
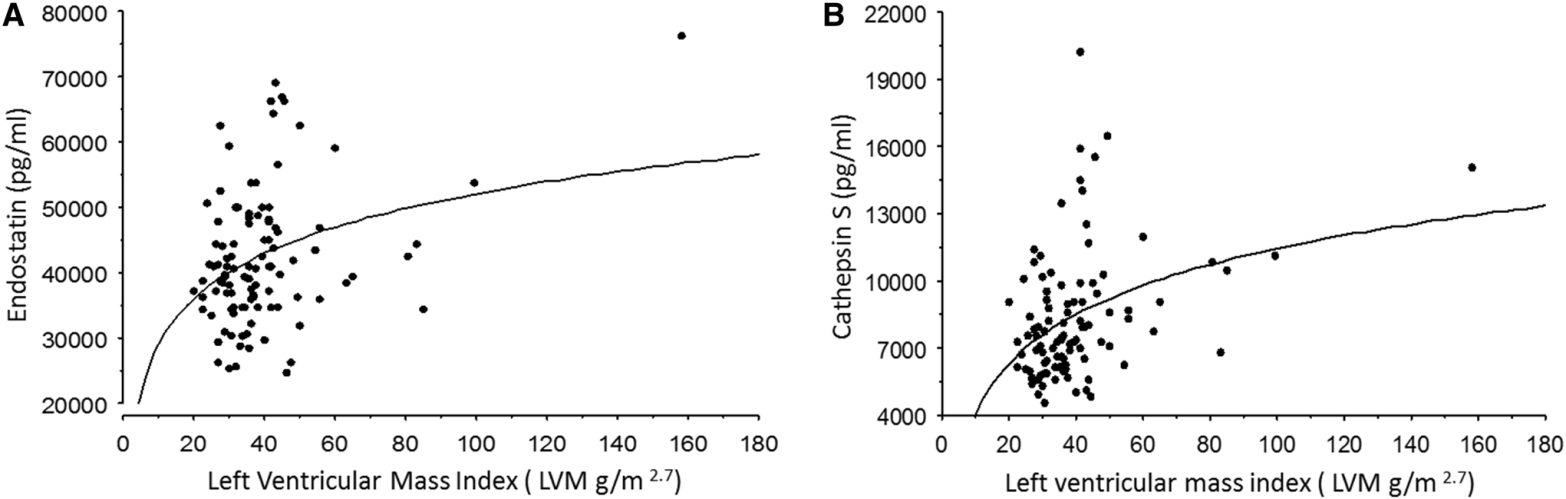
**a** The relationship between endostatin and left ventricular mass index in the study groups (*p* = 0.001; *r* = 0.3). **b** The relationship between cathepsin S and left ventricular mass index in the study groups (*p* = 0.0001; *r* = 0.4).

**Fig. 3:**
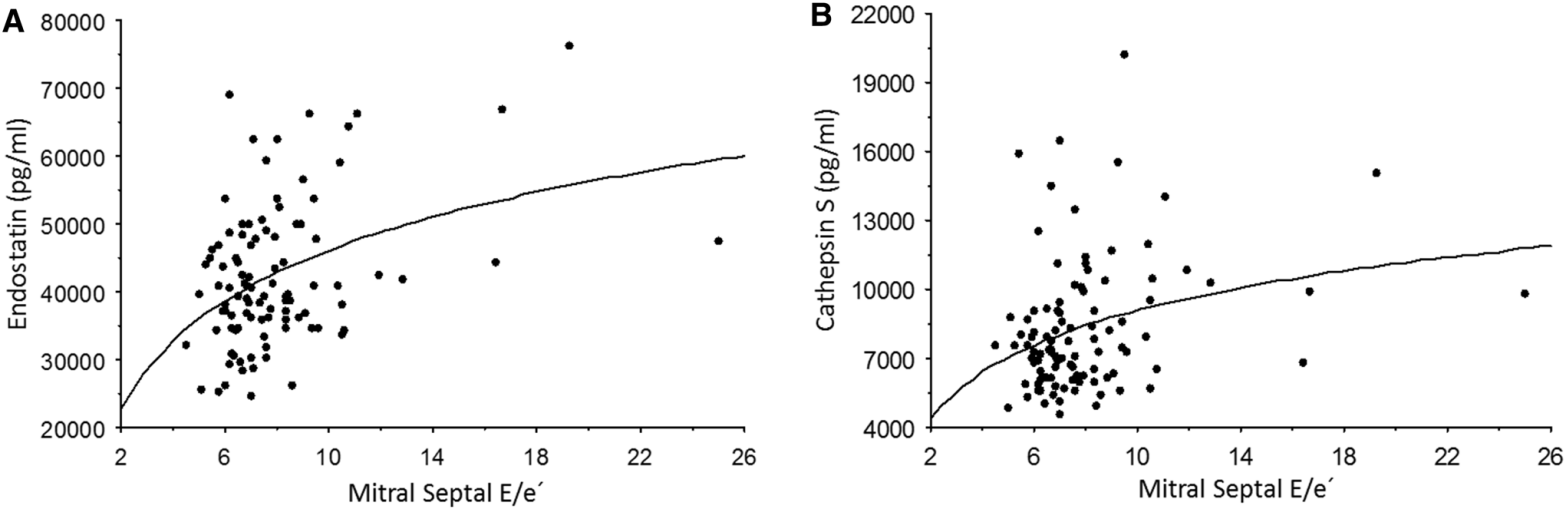
**a** The relationship between endostatin and the mitral septal E/e′ by tissue Doppler (*p* < 0.0001; *r* = 0.4). **b** The relationship between cathepsin S and the mitral septal E/e′ by tissue Doppler (*p* = 0.004; *r* = 0.3).

The biomarker data of the study population are summarized in Table 3. Both endostatin and cathepsin S were increased in the HCM group compared to controls (*p* = 0.0001 and *p* = 0.0009, respectively), athletes (*p* = 0.005 and *p* = 0.0002, respectively), and the HCM-risk group (*p* = 0.005 and *p* = 0.01, respectively; Fig. 1). These two biomarkers correlated with both left ventricular mass index (*p* ≤ 0.001, r ≥ 0.3; Fig. 2) and mitral E/e′ (*p* < 0.005, *r* ≥ 0.3, Fig. 3). Also, MMP-9 (Fig. 1, panel c) and VCAM (*p* = 0.04) were increased in the HCM group compared to controls.

**Table 3:** The absolute values of the biochemical markers in the study groups, data shown as mean ± SEM

	Controls	Athletes	HCM-risk	HCM
Cathepsin S (pg/mL)	7927 ± 346	6711 ± 361	8031 ± 918	10,296 ± 580
Endostatin (pg/mL)	39,595 ± 1202	39,930 ± 1817	42,471 ± 2465	49,730 ± 2757
ICAM (pg/mL)	195,480 ± 8046	220,645 ± 7260	247,531 ± 14,800	178,323 ± 7538
VCAM (pg/mL)	297,296 ± 10,112	332,915 ± 20,117	313,487 ± 23,609	345,852 ± 27,780
MMP-9 (pg/mL)	119,919 ± 7719	137,246 ± 17,804	173,156 ± 25,843	218,871 ± 59,047
Myostatin (pg/mL)	3274 ± 179	3748 ± 511	2062 ± 174	3390 ± 294
VEGFRI (pg/mL)	3897 ± 2413	1892 ± 1200	1175 ± 605	603 ± 101
ICTP (ug/mL)	8.95 ± 0.7	13.9 ± 2.4	12.6 ± 1.5	12.4 ± 3

**Fig. 4:**
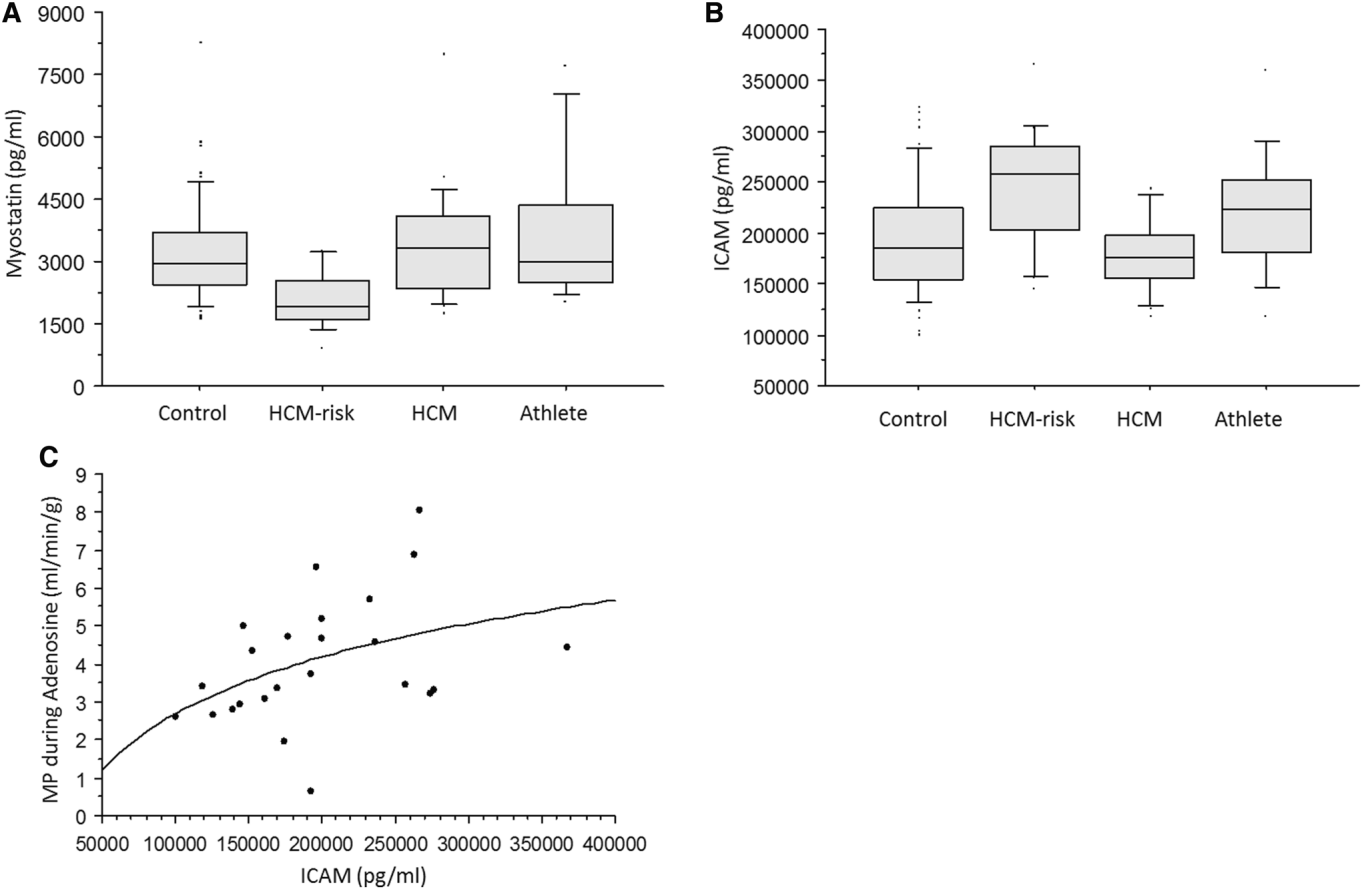
**a** Myostatin was lower in the HCM-risk group than in the other study groups (*p* < 0.01). Data are shown as median and interquartile range. **b** ICAM was higher in the HCM-risk group than in controls (*p* = 0.001) and the HCM group (*p* = 0.0005). Data are shown as median and interquartile range. **c** The relationship between ICAM and the myocardial perfusion (MP) during adenosine hyperemia (*p* = 0.01; *r* = 0.3).

In the HCM-risk group, myostatin was decreased (*p* = 0.004) and ICAM was increased (*p* = 0.002) as compared to the other groups (Fig. 4). ICAM correlated with the myocardial perfusion during adenosine hyperemia, measured by CMR (Fig. 4). There was no significant difference in ICTP or VEGFR1 between the study groups (*p* > 0.1). However, HCM patients with E/e′ > 8 had significantly higher ICTP than the other groups (*p* = 0.02).

### Cardiac Magnetic Resonance

**Fig. 5:**
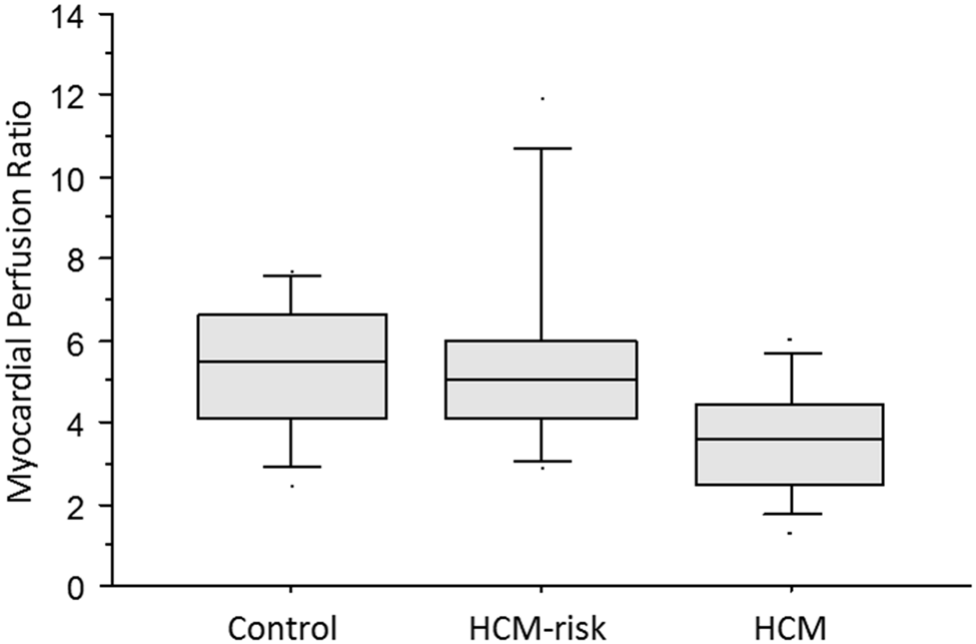
Myocardial perfusion ratio was lower in the HCM group than in the HCM-risk group and controls (*p* = 0.02 for both). No difference in MPR was noted between the HCM-risk group and controls (*p* = 0.7). Data are shown as median and interquartile range.

**Fig. 6:**
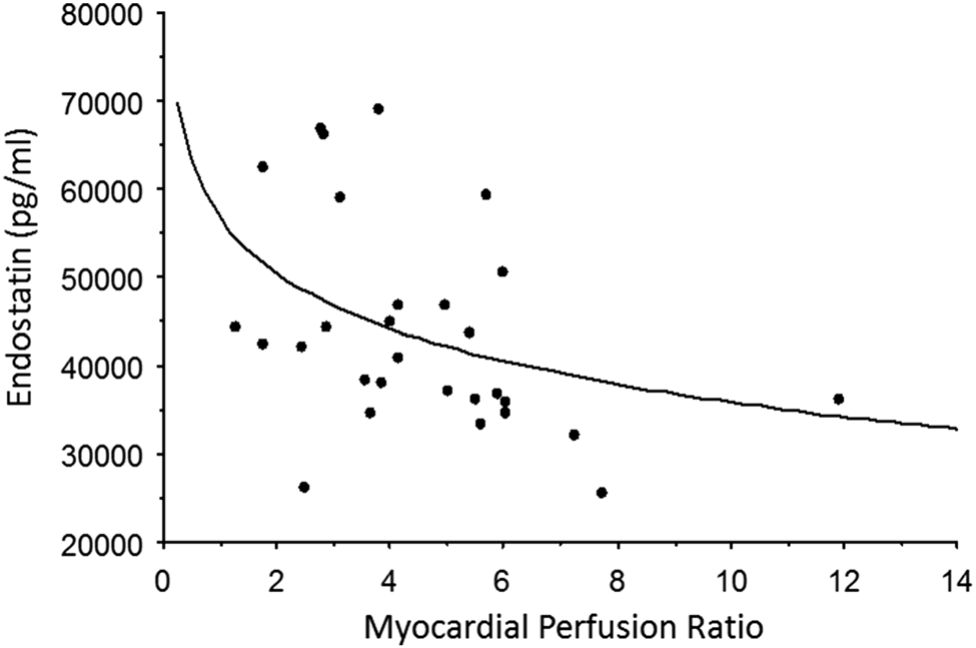
The relationship between endostatin and the myocardial perfusion ratio in the study groups (*p* = 0.046, *r* = − 0.4).

**Fig. 7:**
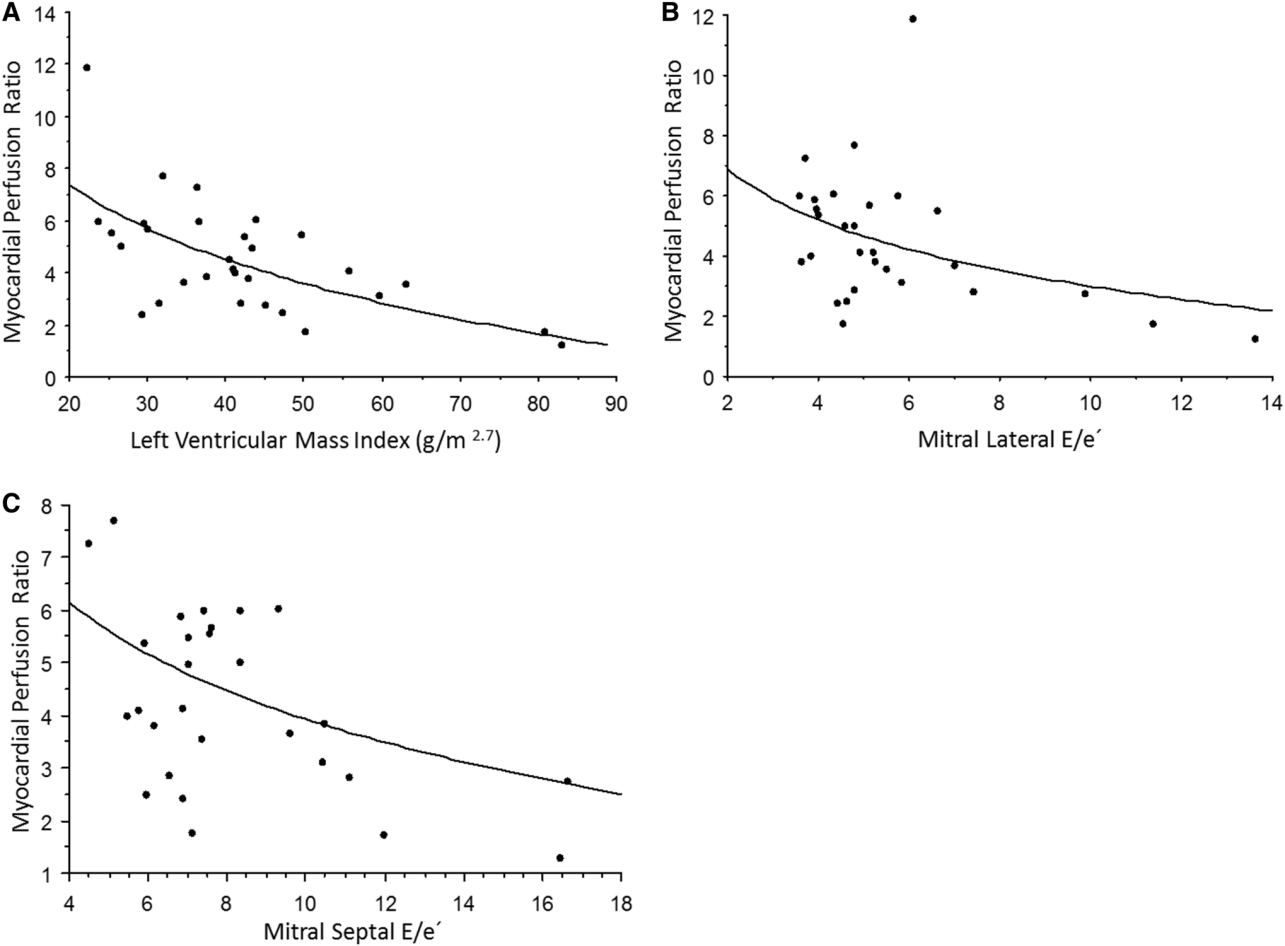
**a** The relationship between the myocardial perfusion ratio and the left ventricular mass index in the study groups (*p* = 0.0002, *r*= − 0.6). **b** The relationship between the myocardial perfusion ratio and the mitral lateral E/e′ in the study groups (*p* = 0.05, *r* = −0.4). **c** The relationship between the myocardial perfusion ratio and the mitral septal E/e´ in the study groups (*p* = 0.05, *r* = − 0.4).

The global myocardial perfusion data are overlapping with the results previously reported [31]. MP was decreased in the HCM group versus controls (*p* = 0.02), even when using *Z* score > 2.5 SD for HCM diagnosis, but no significant changes in MP were found in the HCM-risk group vs. controls (Fig. 5). There was an inverse relationship between serum endostatin and MP during adenosine hyperemia (*p* = 0.04, r = −0.37; Fig. 6). Myocardial perfusion ratio also showed inverse relationship with LVM g/m^2.7^ (*p* = 0.0002, *r* = −0.6), whereas the mitral lateral E/e′ (*p* = 0.05, *r* = −0.4) and septal E/e′ (*p* = 0.004, *r* = −0.5) measured by tissue Doppler correlated inversely to myocardial perfusion change during adenosine hyperemia (Fig. 7).

## Discussion

In the present study of a young cohort of HCM patients and HCM-risk individuals, in whom we earlier have reported abnormalities regarding myocardial diastolic function and peripheral microvascular function [11], we observed significant changes in circulating serum biomarkers reflecting myocardial matrix remodeling and vascular dysfunction. Our findings support the hypothesis that adverse changes in the cardiac extracellular matrix and microcirculation resulting in altered myocardial tissue composition may be identified in the early phase of HCM in the young.

ECM remodeling is probably an early biological process in the development of hypertrophic cardiomyopathy, and is thought to be mediated by proteases, such as cathepsins and MMPs, in response to proinflammatory stimuli and oxidative stress. This process, along with altered coronary angiogenesis, which is in part mediated by endostatin, contributes to myocardial remodeling and development of fibrosis, all of which being considered morphological key features of HCM. Our data indicating elevated levels of circulating cathepsin S and endostatin in the group of children and young adults with HCM corroborate these previous findings [11, 36–38]. In our study, these two biomarkers showed a clear relationship to E/e´, an index of ventricular diastolic function, and to left ventricular mass index (LVM g/m^2.7^). Although we did not find any significant difference in circulating cathepsin S between controls and those at risk for HCM, the association of cathepsin S with these cardiac indices is in agreement with the results of Ho et al. [10] that showed increased myocardial extracellular volume using CMR in individuals with HCM sarcomere mutation but no myocardial hypertrophy [10]. It may also explain our earlier finding of increased mitral E/e´ in individuals at risk for HCM, without noticeable cardiac hypertrophy [11].

In the present study, we observed lower levels of circulating myostatin in the HCM-risk group but not in the HCM group. This could indicate altered myoblast stimulation, a potential mechanism in the early development of myocardial hypertrophy. Myostatin acts through a complex interaction between GH/IGF-1, intracellular SMAD 2/3, protein kinase B (AKT) and mammalian target of Rapamycin (mTOR), which is capable to regulate protein synthesis and cell size [24]. This interaction may possibly explain the very rapid progress of LVH during puberty in HCM patients. Nevertheless, further studies are needed to elucidate the precise role of myostatin in the occurrence and progression of HCM in the young.

There are several recognized serum biomarkers for myocardial fibrosis such as C-terminal propeptide of type I procollagen (PICP), C-terminal telopeptide of type I (ICTP), procollagen III (PIIINP) and N-Terminal Propeptide (PIIINP) [13]. Due to their similarities, we selected only ICTP as a marker of fibrosis. In contrast to a previous report [17], we did not find significant changes of ICTP in our young HCM group or in the HCM-risk group. This could probably be explained by their young age and shorter duration of clinical disease. However, when using mitral E/e´ > 8 as a cut-off for LV diastolic dysfunction, we found higher levels of ICTP (*p* = 0.02) in the HCM group, a finding that could suggest alterations in ECM prior to development of fibrosis. As recently reported [31], the degree of myocardial fibrosis was assessed using CMR -LGE, and the association between regional hypoperfusion and fibrosis [39] in young patients with HCM. In line with previous studies showing a certain correlation between the magnitude of fibrosis and the degree of hypertrophy [2, 7–9], fibrosis was predominantly found in myocardial segments with advanced hypertrophy [39] and also indicating a pathology beyond LVH and LGE by showing greater extent of areas with impaired myocardial perfusion even in non-fibrotic myocardium during adenosine hyperemia indicating regional hypoxemia, suggesting an important relationship of myocardial hypoperfusion with fibrosis occurrence in HCM [39]. Myocardial ischemia is previously suggested as an important complication in HCM [38] and supported by a previous mouse model of HCM [40] where sarcomere mutation-positive animals were found to upregulate genes involved in extracellular matrix remodeling in early stage of the disease, prior to the onset of histological changes characteristic for LVH and fibrosis. A human study [41] recently showed genetic variations in angiogenic and hypoxia-responsive genes with the association to younger age at diagnosis, diastolic dysfunction, and greater septal hypertrophy [41]. Although ICTP showed no correlation with any of the above CMR indices, there was an inverse relationship of myocardial perfusion during adenosine hyperemia with both LVM and mitral E/e′, a finding that strengthens the concept that myocardial perfusion abnormalities occur in parallel with the progression of myocardial hypertrophy and diastolic dysfunction.

Endostatin results from the cleavage of collagen XVIII by proteolytic enzymes such as cathepsins and MMPs [22], and has important antiangiogenic properties by inhibiting endothelial cell proliferation and migration [20, 22, 42, 43]. Our findings showing inverse relationship between CMR myocardial perfusion and endostatin along with elevated circulating levels in the HCM group support the role of endostatin in the defective angiogenesis and possibly also the adenosine-induced hypoperfusion and whereby the regional hypoxemia found in HCM group. Higher levels of endostatin have also previously been shown to be associated with left ventricular mass in a community-based cohort [23] which suggests that endostatin may be a marker of myocardial remodeling even in the absence of HCM.

A prior study suggested a link between circulating endostatin and endothelium-dependent vasomotor dysfunction via decrease in nitric oxide bioactivity [42]. We have noticed abnormal microvascular response to acetylcholine, an endothelium-dependent agonist, in the peripheral circulation in both the HCM and HCM-risk groups [11]. Myostatin may also exert coronary and systemic effects on vascular function via inhibition of eNOS phosphorylation and increase in ICAM-1 and VCAM-1 expression [44]. The latter could explain the elevated levels of ICAM and VCAM in the HCM-risk and HCM groups, respectively.

### Study Limitations

HCM is a very rare disease in the young, which explains in part the small size of the HCM and HCM-risk cohorts. In addition, not all HCM probands were positive for known HCM mutations, but they all fulfilled the echocardiographic criteria for HCM and all had a family history for HCM. The HCM-risk group was also younger than the other groups. Another potential concern is biased results due to “common family genetics.” However, the individuals in the HCM and HCM-risk groups were recruited from 26 unrelated families participating in these two groups. Intuitively, this would have minimized any such bias.

In conclusion, the present study indicates altered circulating biomarkers of myocardial extracellular matrix remodeling and vascular endotheliopathy in the early phase of HCM in the young. The findings suggest an interesting pathophysiological biochemical process in early stages of HCM and in individuals at risk for developing this disease. Further studies are warranted to confirm this hypothesis and to assess whether they can be used to estimate the risk for HCM-related complications in the later phase of the disease. Whether these early biochemical changes reflecting altered myocardial tissue composition in HCM could add further information in risk stratification of individuals with HCM-causing mutation remains to be established.
